# Investigation of Pectenotoxin Profiles in the Yellow Sea (China) Using a Passive Sampling Technique

**DOI:** 10.3390/md8041263

**Published:** 2010-04-15

**Authors:** Zhaoxin Li, Guo Mengmeng, Yang Shouguo, Wang Qingyin, Tan Zhijun

**Affiliations:** 1 Yellow Sea Fisheries Research Institute, No.106 Nanjing Road, 266071 Qingdao, China; 2 Norwegian School of Veterinary Science, P.B. 8156, 0033 Oslo, Norway; 3 College of Fisheries and Life Science, Shanghai Ocean University, 200090, Shanghai, China

**Keywords:** pectenotoxin, mussel, mytilus edulis, LC-MSMS, SPATT, passive sampling

## Abstract

Pectenotoxins (PTXs) are a group of lipophilic algal toxins. These toxins have been found in algae and shellfish from Japan, New Zealand, Ireland, Norway and Portugal. PTX profiles vary with geographic location of collection site. The aim of the present study was to investigate PTX profiles from the Yellow Sea, China. The sampling location was within an aquatic farm (N36°12.428′, E120°17.826′) near the coast of Qingdao, China, in the Yellow Sea from 28 July to 29 August 2006. PTXs in seawater were determined using a solid phase adsorption toxin tracking (SPATT) method. PTXs were analyzed by HPLC-MSMS. PTX-2, PTX-2 *sec* acid (PTX-2 SA) and 7-*epi*-PTX-2 SA were found in seawater samples. The highest levels of PTXs (107 ng/g of resin PTX-2, 50 ng/g of resin PTX-2 SA plus 7-*epi*-PTX-2 SA) in seawater were found on 1 August, 2006. From 1 August to 29 August, the levels of PTX-2 and PTX-2 SA decreased. In the same area, the marine algae, *Dinophysis acuminata* was found in the seawater in the summer months of 2006. This indicated that *Dinophysis acuumuta* might be the original source of PTXs. PTX-11 and PTX-12a/b were not found in seawater.

## 1. Introduction

**Figure 1:**
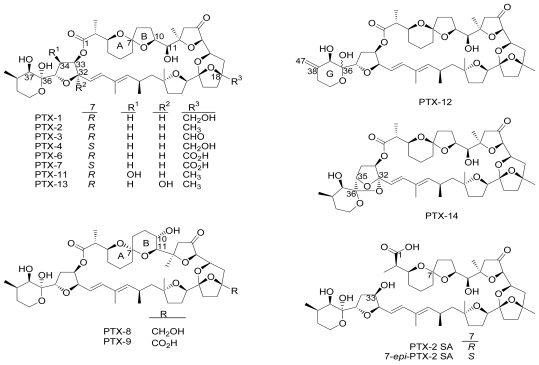
Structures of PTX-1,2,3,4,6,7,8,9,11,12,13,14, PTX-2 SA and 7-*epi*-PTX-2 SA [14].

Pectenotoxins (PTXs) are a group of lipophilic toxins found in dinoflagellates *Dinophysis fortii*, *D. acuta*, *D. acuminata*, *D. caudata* and *D. norvegica* [1–10]. PTX-1 and PTX-2 were first isolated in 1985 from Japanese scallops *Patinopecten yessoensis* [10]. PTXs are cyclic polyether-lactone rings. The conversion of PTX-2 into its analogues occurs in bivalve shellfish [10–13]. The chemical structures of PTX-1, 2, 3, 4, 6, 7, 8, 9, 11, 12, 13 and 14, as well as their seco acids, PTX-2 SA and 7-*epi*-PTX-2 SA, have been confirmed (Figure 1) [14]. Chemical structures of PTX-5 and PTX-10 have not yet been reported [15].

There is no evidence of adverse acute health effects of PTXs in humans. In many countries there are currently no regulatory procedures or guidelines for the quantification of PTX toxins alone in shellfish, but the limit of PTXs together with other toxins in the DSP group has been established. The European Commission has established the following guidelines: maximum level of OA, DTXs and PTXs together, in edible tissues (whole body or any part edible separately) of molluscs, echinoderms, tunicates and marine gastropods shall be no more than 160 mg OA equivalents/kg [16,17]. The mouse bioassay established by Yasumoto *et al.* is adopted as a reference method [18]. The investigation of the geographic distribution and toxin profiles of PTXs is important. The toxin profiles of seawater, algae and bivalve shellfish have a close relationship. Algae produce and release toxins in seawater when the cells are broken. Bivalve shellfish accumulate toxins by filtering toxic algae.

The concept for the development of solid phase adsorption toxin tracking (SPATT) comes from the observation during natural blooms and culture studies on toxic algae [19–21]. The discs which contain absorbent material can accumulate the parent toxins that are released by toxic algae in the seawater. In the investigation of PTXs in seawater, the absorption efficiency of HP-20 resin shows that it can accumulate significantly greater amount of lipophilic toxins than the other resins, such as SP 825, SP 850, L 493 and XAD 4 [21]. Rundberget *et al*. have developed a large–scale method for solid-phase extraction of lipophilic biotoxins from natural microalgal blooms in seawater [19,20]. Isolation of OA, DTX-2 adsorbed on the SPE column (HP-20 resin) was simple and efficient, and Rundberget *et al* [20] were able to extract OA and DTX-2 standards from seawater using this technique. The experimental results also indicated that the method was potentially applicable to a wide range of other microalgal toxins such as azaspiracids, spirolides and microcystins. SPATT technique has also been used to forecast the potential contamination of toxins in shellfish in many countries [19–22].

Pectenotoxin profiles in Chinese waters have not been previously reported. In 2006 SPATT discs were given from Norwegian National Veterinary Institute (NVI) as gifts to investigate the toxin contents in seawater from Qingdao Bay, the Yellow Sea, China. In this area the main aquiculture species include scallop *Chlamys farreri*, oyster *Crassostrea gigas*, blue mussel *Mytilus edulis* and Manila clam *Ruditapes philippinarum*. Usually scallops and blue mussels are in raft culture. Oysters and Manila clams are cultured in the bottom sowing.

## 2. Results and Discussion

### 2.1. PTX standard chromatograms

**Figure 2:**
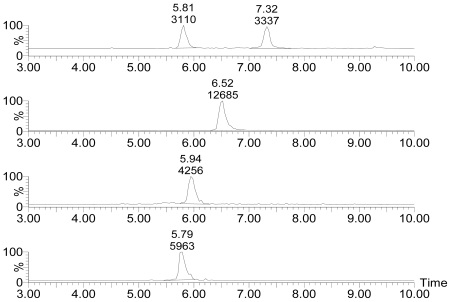
Selected ion channel *m/z* 874.5 → 821.4 (A), 876.5 → 823.4 (B), 892.5 → 839.4 (C), 894.5 → 823.4 (D) chromatograms of standards of PTX-12a and PTX-12b (reference mussels), PTX-2 (40 ng/mL), PTX-11 (40 ng/mL) and PTX-2 SA (40 ng/mL).

Figure 2 displays the selected ion channel *m/z* 874.5 → 821.4, 876.5 → 823.4, 892.5 → 839.4, 894.5 → 823.4 chromatograms of standards of PTX-12a/b, PTX-2, PTX-11 and PTX-2 SA, respectively. The retention time (r.t.) of standard materials of PTX-12a, PTX-12b (Figure 2A) and PTX-11 (Figure 2C) were 5.81 min, 7.32 min and 5.94 min, respectively. The retention time of the standards were used in the qualitative analysis of PTXs in seawater. The calibrations linear range of standards of PTX-2 (Figure 2B, r.t. = 6.52 min) and PTX-2 SA (Figure 2D, r.t. = 5.79 min) was from 0 to 40 ng/mL. If the concentration of PTX-2 or PTX-2 SA in the disc eluents was higher than 40 ng/mL it was diluted to a suitable level with 80% methanol before being re-injected into the LC-MSMS system.

### 2.2. PTXs in seawater

**Figure 3:**
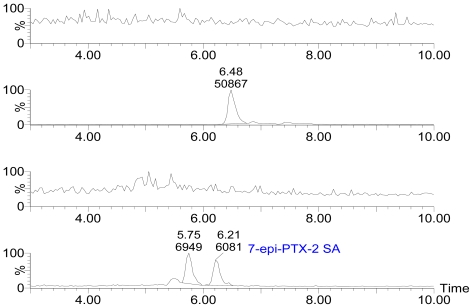
Selected ion channel m/z 874.5 → 821.4 (A), 876.5 → 823.4 (B), 892.5 → 839.4 (C), 894.5 → 839.4 (D) chromatograms of PTX-12a/b, PTX-2, PTX-12 SA, PTX-2 SA and 7-*epi*-PTX-2 SA, respectively, in sea water.

**Figure 4:**
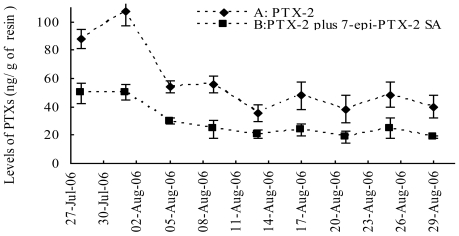
Time series of observed levels of PTX-2 (A), PTX-2 SA plus 7-*epi*-PTX-2 SA (B) in seawater at 6 m depth at from the Yellow Sea, China (N36° 12.424′, E120° 17.826′) during the period 27 July 2006 to 29 August 2006. The errors bars are standard deviation of “n” SPATT, n = 3.

Figure 3 displays the selected ion channel m/z 874.5 → 821.4 (A), 876.5 → 823.4 (B), 892.5 → 839.4 (C), 894.5 → 839.4 (D) chromatograms of PTX-12a/b, PTX-2, PTX-12 SA, PTX-2 SA and 7-*epi*-PTX-2 SA, respectively, in extracts of seawater on August 1, 2006. The levels of PTXs in seawater were investigated from July 28 to August 29, 2006. Figure 4 shows the variation of PTX levels during the evaluation period.

PTX-12a/b and PTX-11 were not found in any of the adsorption resin disc extracts (Figure 3A, 3C). The result showed that PTX-2, PTX-2 SA and 7-*epi*-PTX-2 SA are present in the Yellow Sea (Figure 3B, 3D). 7-*epi*-PTX-2 SA is the result of interconversion of PTX-2 SA, and the latter is more thermodynamically stable [13]. Thus, the concentration of 7-*epi*-PTX-2 SA in seawater was calibrated by comparing with the standard of PTX-2 SA. The total amount of PTX-2 seco acid was expressed by PTX-2 SA plus 7-*epi*-PTX-2 SA. In July the levels of PTX-2 (88.0 ± 6.8 ng/g of resin, n = 3) and PTX-2 SA plus 7-*epi*-PTX-2 SA (49.5 ± 7.2 ng/g of resin, n = 3) in seawater were relatively high. On August 1 the highest levels of PTX-2 (107.3 ± 10.0 ng/g of resin, n = 3) and PTX-2 SA plus 7-*epi*-PTX-2 SA (50.2 ± 5.7 ng/g of resin, n = 3) were found. From 1 August to 9 August the levels of PTXs decreased relatively quickly. From 9 August to the end of August the variation in PTX levels was small. On 29 August, the levels of PTX-2 and PTX-2 plus 7-*epi*-PTX-2 SA were 40.0 ± 7.9 ng/g, n = 3, of resin and 18.5 ± 1.0 ng/g, n = 3, of resin, respectively; which is about the one-third of the highest levels (Figure 4). During the evaluation period the range of PTX-2 concentration in seawater was from 40 to 107 ng/g of resin. Compared with the concentration of PTX-2 (100–1,500 ng/g of resin) in seawater at a monitoring location at Wedge Point, Queen Charlotte Sound, New Zealand [23], the levels of PTXs in seawater from the Yellow Sea were relatively lower.

Pectenotoxins (PTXs) are a group of lipophilic algal toxins. It is important to study the geographic distribution of marine toxins. PTXs have been found in algae or shellfish in Japan, Ireland, New Zealand, Norway, Italy, Spain, Portugal [4–6,13,15,24]. Along the coast of the Yellow Sea China, they have never been reported. The SPATT technique was first described by MacKenzie *et al.* as a technique to monitor the occurrence of toxic algal blooms and shellfish contamination events [23]. This technique is an effective method for the investigation of lipophilic marine toxins in seawater [19,20,23]. It is simple, economical and efficient. The monitoring results can show the toxins produced by algae, avoid derivatives occurring in shellfish [21].

The Diaion HP-20 resin has been identified as a suitable resin for effectively accumulating lipophilic toxins from seawater [21]. Furthermore, the HP-20 resin has been used to adsorb a large amount of OA and DTX-2 from seawater in Spain and Norway [19,20]. In agreement with other experiments [21,23,25], the HP-20 resin was selected as the absorbent in the investigation of PTX profiles in the Yellow Sea. The HP-20 is a porous polystyrene adsorbent resin which, by its relatively large pore sizes (260 Å), makes it suitable for recovery of algal toxins that can be eluted with common organic solvents. Experiments showed HP-20 could also accumulate OA and DTX-1 linearly up to 72 h contact time [21].

In our experiment, the SPATT discs which contained HP-20 resin were immerged in seawater for a period of 4 days. In the field trials of toxin monitoring with SPATT techniques, the discs were generally replaced weekly in New Zealand [23]. The immerged time of SPATT discs in seawater might affect the amount of toxins adsorption onto the HP-20 resin, but to date there are no agreed guidelines for immerged time of the discs in seawater. Though the SPATT technique has showed its advantage as a tool used in the investigation of lipophilic toxins in seawater, it still needs to be developed. One of the problems waiting to be solved is to derive the absolute concentration of lipophilic toxins in seawater. Many studies which used SPATT technique reported the relative concentration of lipophilic toxins in seawater using the unit ng toxin/SPATT bag [23] or μg/g of resin [21].

### 2.3. The algal source of PTXs

It was revealed that PTXs originated from *D. fortii*, *D. acuta*, *D. acuminate* in Japan, New Zealand, Norway, Italy, Spain and Portugal [4,6,13,15,24]. Living toxic algae can produce PTXs which can be released into seawater. OA, DTXs and PTXs are always found together in New Zealand *Dinophysis* spp. [6]. Zhou *et al.* [26] reported the distribution of DSP along the Chinese coast, but few studies on PTXs have been reported in China. In the present study PTX-2, PTX-2 SA and 7-*epi*-PTX-2 SA were detected in all extracts of SPATT discs from 28 July to 29 August, 2006 (Figure 4). Toxic algae were not investigated in parallel with this study, but, from May to July 2006, another Chinese research group revealed the existence of *D. acuminata* in this bay (H. Xianqin, Yellow Sea Fishery Research Institute, personal communication). *D. acuminata* may be the source of PTXs. Up to now PTX-12a/b has only reported from *D. acuta, D. Norvegica* and *D. rotundata* from the Scandinavian Sea [13]. PTX-11 is found mainly in *D. acuta* from New Zealand [27]. PTX-12a/b and PTX-11and their original algal sources were not found in coastal waters of the Yellow Sea. Further investigation into the algal source and the seasonal variation of PTXs, in this bay is recommended.

## 3. Experimental

### 3.1. Reagents, PTX standards and toxin absorption discs

HP-20 (Diaion HP-20, 260Å, Mitsubishi Chemical Corporation) was selected as the absorbent resin. Methanol, acetonitrile were of HPLC grade, formic acid and ammonium formate were of analytical-regent grade. Both were obtained from Rathburn Chemical (Walkerburn, UK). Water used was deionized (MilliQ). Standards of PTX-2 [28], PTX-2 SA [27] were obtained from NRC Canada. Standard material PTX-12a/b [29] and PTX-11 were purified in NVI. The discs which were produced in NVI (8 cm in diameter) contained the absorbent resin (3.0 g HP-20) between two layers of plankton net (40 μm in mesh diameter). Totally 27 discs were prepared. Before being immerged in seawater, the discs were activated by placing the frame into a beaker filled with methanol for 15 min, then transferred into a beaker filled with water. The water was renewed three times at 5 minute intervals [19].

### 3.2. PTX extraction from seawater

SPATT techniques were used to adsorb toxins from seawater [19–23]. Each time, three discs were attached to a scallop basket that was 6 m beneath sea level at the pointed sampling location (N36°12.428′, E120°17.826′). The discs were replaced by new ones at four day intervals. Extraction of the toxins from the resin was performed following the method from Fux *et al.* [21] with modifications by Rundberget *et al.* [19]. The discs were rinsed with distilled water and kept in the already marked plastic bags or stored at 4 °C until further preparation. The resin was quantitatively transferred into an empty column cartridge fitted with a nylon frit (Varian) and washed free of salts with 30–50 mL of deionized water. Excess water was drawn out of the column prior to application of 10 mL of methanol. After addition of methanol, the column material was stirred and left static for 15 min before the column was eluted slowly into a rotary flask. This was repeated with another 10 mL of methanol. After elution, an additional 3 mL of methanol was pushed through the column to wash out the remaining dead volume of solvent. The methanol fraction was evaporated to dryness, and redissolved in 1.0 mL 80% methanol. All samples were diluted to appropriate concentrations with 80% MeOH. The 80% methanol extraction was filtered through a 0.22 μm Costar Spin-X^®^ centrifuge filter and transferred into auto-sampler vials. A 10 μL aliquot of the extract was analyzed on the LC-MS-MS system.

### 3.3. Quantitative liquid chromatography mass-mass spectrometry

For the determination of PTXs in the extracts of seawater, a HPLC-MS-MS method following Quilliam was adopted [28,30]. LC-MS-MS spectrometry was performed using a Waters Alliance 2695 LC-system coupled to a Micromass QuattroUltima triple quadrupole mass spectrometer. Data collection was performed using MassLynx v4.0 software. The MS system was equipped with an electrospray ion source (ESI) operating in positive ionization mode. Injection aliquots were separated on a Waters XTerra C18 column (50 × 2.1 mm, 3.5 μm) connected to a C_18_ pre-column (10 × 2.1 mm, 3.5 μm). The mobile phases consisted of 2 mM ammonium formate and 50 mM formic acid in 95% acetonitrile (mobile phase A) and 2 mM ammonium formate and 50 mM formic acid in water (mobile phase B). A gradient was run at 0.4 mL/min from 35% A to 100% A for 11 min, followed by an isocratic period for 5.5 min at 100% A. The gradient was returned to 35% A over 0.5 min and left to equilibrate at 35% A for 3 min before the next injection [28,30]. The ion source was operated at a temperature of 100 °C, using a capillary voltage of 3.5 kV, a cone voltage of 80 V and a cone gas flow of 98 L/hr. The desolvation temperature was set at 250 °C and desolvation gas flow at approximately 510 L/hr. Chromatograms were recorded for 13 min (from 2 min to 15 min). MS recording parameters were as follows: for determination of PTX-2, PTX-2 SA, PTX-11, PTX-12, MRM-chromatograms were recorded for the transitions *m/z =* 876.5 → 823.5 (PTX-2), *m/z =* 894.5 → 823.5 (PTX-2 SA and 7-*epi*-PTX-2 SA), *m/z =* 874.5 → 821.5 (PTX-12a/b), *m/z =* 892.5 → 839.5 (PTX-11). The concentration of PTX-2 SA and 7-*epi*-PTX-2 SA were calculated by making a calibration curve for PTX-2 SA standard and comparing peak areas for PTX-2 SA and 7-*epi*-PTX-2 SA with that for PTX-2 SA. PTX-2 was calculated by making a calibration curve for PTX-2 standard and comparing its peak area with that for PTX-2. PTX-12a, PTX-12b and PTX-11 were determined by comparing the retention time with that for PTX-12a, PTX-12b and PTX-11 reference material.
